# The Economic Burden of Cancer in Canada from a Societal Perspective

**DOI:** 10.3390/curroncol29040223

**Published:** 2022-04-14

**Authors:** Roxanne Garaszczuk, Jean H. E. Yong, Zhuolu Sun, Claire de Oliveira

**Affiliations:** 1 The Canadian Partnership Against Cancer, Toronto, ON M5H 1J8, Canada; roxanne.garaszczuk@partnershipagainstcancer.ca (R.G.); zhuolu.sun@partnershipagainstcancer.ca (Z.S.); claire.deoliveira@york.ac.uk (C.d.O.); 2 Institute of Health Policy, Management & Evaluation, Dalla Lana School of Public Health, University of Toronto, Toronto, ON M5T 3M7, Canada; 3 Centre for Health Economics and Hull York Medical School, University of York, Heslington, York YO10 5DD, UK; 4 Centre for Addiction and Mental Health, Institute for Mental Health Policy Research and Campbell Family Mental Health Research Institute, Toronto, ON M6J 1H4, Canada

**Keywords:** economic burden, societal perspective, cancer, health system’s costs, out-of-pocket costs, time costs, indirect costs

## Abstract

Cancer patients and their families experience considerable financial hardship; however, the current published literature on the economic burden of cancer at the population level has typically focused on the costs from the health system’s perspective. This study aims to estimate the economic burden of cancer in Canada from a societal perspective. The analysis was conducted using the OncoSim-All Cancers model, a Canadian cancer microsimulation model. OncoSim simulates cancer incidence and deaths using incidence and mortality data from the Canadian Cancer Registry and demography projections from Statistics Canada. Using a phase-based costing framework, we estimated the economic burden of cancer in Canada in 2021 by incorporating published direct health system costs and patients’ and families’ costs (out-of-pocket costs, time costs, indirect costs). From a societal perspective, cancer-related costs were CAD 26.2 billion in Canada in 2021; 30% of costs were borne by patients and their families. The economic burden was the highest in the first year after cancer was diagnosed (i.e., initial care). During this time, patients and families’ costs amounted to almost CAD 4.8 billion in 2021. This study provides a comprehensive estimate of the economic burden of cancer, which could inform cost–benefit analyses of proposed cancer prevention interventions.

## 1. Introduction

Two in five Canadians are expected to develop cancer in their lifetime [1]. Furthermore, cancer treatment costs have increased significantly over recent years, nearly doubling for some cancer sites [2]. The rising costs of cancer have been associated with medical debt and even bankruptcy among people in the United States [3]. In Canada, a country with universal public health care insurance, people with cancer still incur serious personal costs; thus, it is important to have a good understanding of the economic burden of cancer [4]. Cost-of-illness studies translate the adverse effects of cancer into dollars, which can be helpful to advocate for more treatment and support as well as provide input for decision making around prevention interventions. Existing published literature on the economic burden of cancer at the population level has generally focused on the burden from the public healthcare payer’s perspective (i.e., direct health system costs) [5]. However, this approach only covers one component of the total economic burden of cancer. It is also important to understand the economic burden from the perspective of patients and their families, which can be considerable [6,7,8]. Studies that measure the economic burden of cancer from the perspective of patients and their families usually measure limited categories of costs (e.g., out-of-pocket cost or lost earnings) for specific types of patients (e.g., long-term prostate cancer survivors or colorectal cancer patients receiving palliative care) [6,7,8]. Moreover, costs are often reported on a per-patient basis or per month, which may not be comparable to other estimates of the economic burden, which are reported as annual costs at the population level [9]. A thorough understanding of the economic burden of cancer will help inform cancer-related policies intended to support cancer patients and their caregivers. The objective of this study was to provide a comprehensive estimate of the economic burden of cancer in Canada from a societal perspective (i.e., the perspective of the health care system and patients and their families) using a cancer microsimulation model and published literature.

## 2. Materials and Methods

### 2.1. OncoSim Model Overview

The analysis was conducted using OncoSim (version 3.4.2.9), a web-based Canadian cancer microsimulation tool co-developed by Statistics Canada and the Canadian Partnership Against Cancer. This analysis used the OncoSim-All Cancers model, one of the five OncoSim modules, which simulates the incidence and deaths of 31 cancers: oral cavity, oropharynx, hypopharynx, other oral, esophagus, stomach, colorectal, liver, pancreas, larynx, lung, melanoma, breast, cervix, uterus, ovary, prostate, testis, bladder, kidney, brain/central nervous system, thyroid, Hodgkin’s lymphoma, non-Hodgkin’s lymphoma, multiple myeloma, leukemia, vulva, vagina, anal, penile and others.

**Figure 1:**
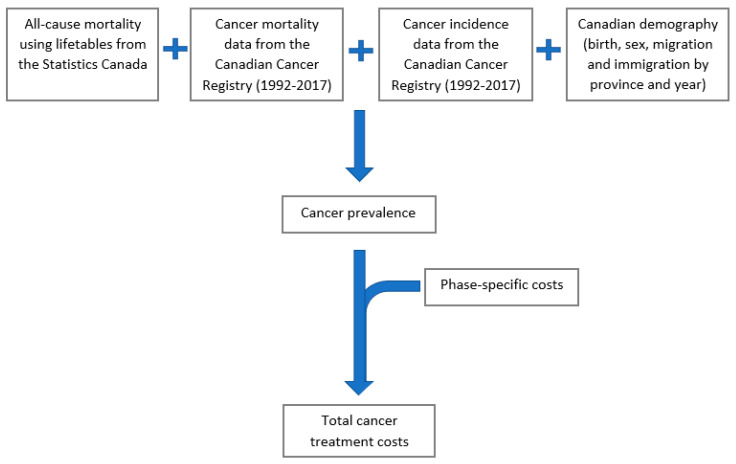
Schematic diagram of OncoSim-All Cancers model.

OncoSim simulates the Canadian population using historic (observed) and projected demographics available from Statistics Canada, including birth, migration, and mortality by age, sex, year, and province/territories. OncoSim simulates one person at a time, estimating whether and when an individual will develop cancer and whether this individual will die from cancer or other causes (Figure 1).

**Figure 2:**
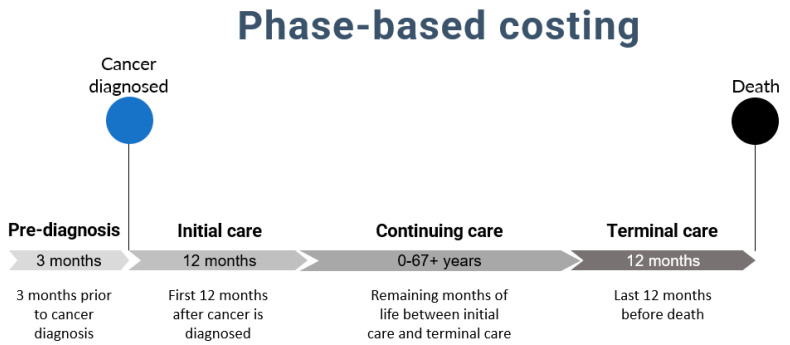
Definition of care phases in phase-based costing method.

In addition, for each person diagnosed with cancer, the model estimates cancer treatment costs using a phase-based costing method (Figure 2), commonly used for estimating costs of cancer care [10,11]. The duration of the phases of care was determined from previous literature on costs of cancer which, in turn, was based on clinical knowledge of the disease and patterns in the data [10,12,13]. Figure 2 provides the definition and duration of care phases according to the phase-based costing method. For example, in the model, if a person died from cancer 10 years after a cancer diagnosis, this person would incur costs during the pre-diagnosis phase (3 months), the initial phase of care (12 months after diagnosis, including date of diagnosis), the continuing phase of care (the remaining months of life between the initial and terminal phases of care, i.e., 8 years in this case) and the terminal phase of care (last 12 months before death-which may or may not be due to cancer).

### 2.2. Conceptual Framework

**Figure 3:**
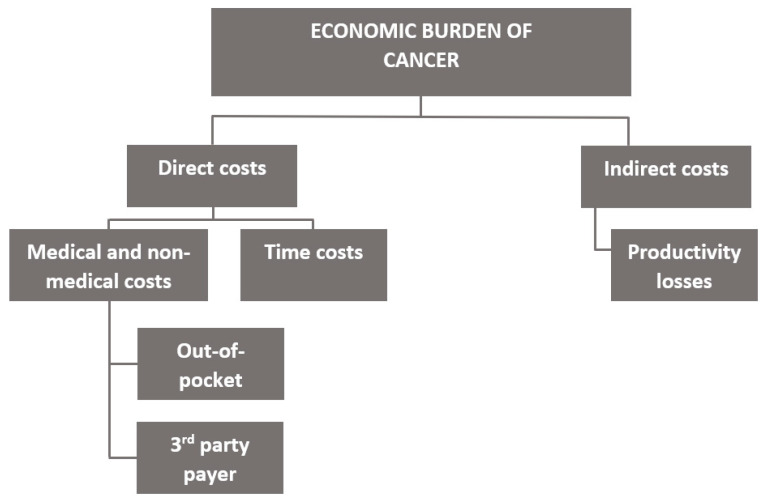
Conceptualization of the economic burden of cancer. Adapted from Pisu et al. diagram [14].

The economic burden of cancer was estimated from a societal perspective following Pisu et al.’s framework (see Figure 3) [14] and included the following costs based on data availability: direct medical costs from the third-party public healthcare payer (health system), direct out-of-pocket costs (medical and non-medical costs), direct time costs, and indirect costs (from the patient perspective). While the true societal perspective would have included donations from charity organizations (i.e., funds used to subsidize patients’ cancer-related expenses), costs from the private third-party payer, psychosocial costs (i.e., quality of life costs), and indirect costs of caregivers, we did not include these in our base case estimates because data on these costs were limited. Direct health system costs included costs of services provided in hospitals and by physicians as well as some prescription drugs. Out-of-pocket costs (OOPCs) included costs of drugs/supplements, home care, equipment, and all other expenses incurred by the patient. Time costs included the cost of the time to travel to obtain care, wait for care, and receive care. Indirect costs included lost earnings from employment from the patient’s perspective. The term “patients’ and families’ costs” is used to refer to out-of-pocket, time, and indirect costs, collectively.

### 2.3. Data Sources

The net medical health system costs of cancer procedures and management were obtained from a study that estimated the net costs of cancer care using a matched case–control study design using administrative health care data from Ontario [10]. Net costs (i.e., costs due to cancer) were obtained by subtracting the costs incurred by cancer patients from those incurred by matched controls. This study examined adult patients diagnosed with a primary cancer between 1997 and 2007 and who survived 30 days or more. The net health system costs included costs associated with chemotherapy, radiation therapy, all physician services (including diagnostic tests and laboratory services), inpatient hospitalizations, ambulatory care (including same-day surgeries/procedures and emergency department visits), other institution-based care (such as long-term care and complex continuing care) and home care [10]. The net health system costs also included outpatient prescription drugs for: patients aged 65 and over, those living in a long-term care home, home for special care, and community home for opportunity, patients aged 24 and younger who are not covered by a private insurance plan, individuals receiving professional home and community care services, individuals receiving benefits from Ontario Works or Ontario Disability Support Program, and those enrolled in the Trillium Drug Program [10]. The study reported net health system costs by cancer site, phase of care (pre-diagnosis, initial, continuing, and terminal), and sex.

The gross OOPCs by phase of care were obtained from three studies. First, out-of-pocket costs incurred during the first 12 months after diagnosis (i.e., initial care) were retrieved from a longitudinal study that surveyed patients diagnosed with breast, colorectal, lung, and prostate cancers with a minimum of 4 weeks of cancer treatment in Canada [9]. The costs were self-reported and included costs of drugs, homecare, alternative medicines, supplements, accommodations, devices, family care, travel, parking, and other costs. Second, OOPCs for the continuing care months were obtained from a cross-sectional study that surveyed long-term prostate cancer survivors diagnosed in 1993–1994, 1997–1998, and 2001–2002 in Ontario [15]. Here, the OOPCs included expenses spent on visiting health professionals, medication, equipment, community services, household help, and time lost from work and leisure. Although this study only examined one cancer site, we assumed this study would best represent continuing care costs. During the continuing care phase, many cancer patients were survivors and would, therefore, be spending less money on cancer-related products than those actively receiving treatment in the initial or terminal phases of care. For the two studies which reported OOPCs for select cancers (i.e., breast, colorectal, lung, prostate), averages of the reported cancer sites were used for all other sites. Lastly, OOPCs incurred during the 12 months before death (i.e., terminal care) were obtained from a prospective longitudinal study that estimated personal costs related to palliative care from cancer patients enrolled in a regional palliative care program in Canada [16]. Note, in the OncoSim model, not all individuals who die of cancer were in palliative care.

We estimated the net direct time costs by multiplying the net number of hours receiving care by the median hourly wage in Canada in 2021 (CAD 19.14) [17]. The net number of hours of care were obtained from a nested case–control study from the US that compared the time receiving care for individuals aged 65 years or older diagnosed with colorectal cancer and those without, by phase of care [12]. On average, patients spent 302 h in the initial care phase, 183 h in the terminal care phase, and approximately 20 h annually in the continuing care phase.

Net indirect costs associated with cancer (i.e., patients’ lost earnings from employment) were obtained from a matched case–control longitudinal study, which linked data from the Canadian Cancer Registry, the Vital Statistics Registry, and personal income tax records [18]. The study included all cancer patients aged 25 to 61 years who survived at least 3 years after diagnosis and reported estimates separately for each year after diagnosis. The costs from the first year after diagnosis were used as estimates of the indirect costs for the initial care phase, while an average of the costs for the second and third years after diagnosis were used to obtain estimates of the indirect costs for the continuing care phase. One advantage to this study was that the authors reported indirect costs by high, medium, and low survival cancers. Unfortunately, there were no high-quality studies, which presented indirect costs for the terminal phase of care. For this reason, the previous estimates of the initial care phase were also used for the terminal care phase in the model. The rationale behind this is that during the terminal phase of care patients receive end-of-life and/or palliative care, and thus are not likely working. Therefore, it is probable that both phases will have similar periods of absence from the labour market [19,20].

All original cost estimates from the studies selected were converted to 2021 Canadian dollars using the Consumer Price Index (CPI) for All Items [21]. In addition, cost estimates were converted to a 12-month cost estimate, where required (Table 1). For example, in studies that reported costs for 3 months, we assumed this value would be the same for the remaining 9 months of the year (3-month cost × 4 = 12-month estimate). Refer to Supplementary Table S2 for details on cost conversions.

### 2.4. Assumptions

Several assumptions were required to undertake the analysis, specifically for the continuing phase of care. The OncoSim model is designed to continuously project cancer costs as long as an individual is alive; thus, if an individual becomes cancer-free or has returned to work, costs will still be projected during that time. To prevent overestimating continuing care costs, we first assumed that cancer patients received additional medical care due to cancer for a maximum of 10 years during the continuing care phase because 10 years was the longest observation period from the source study [10]. Second, we assumed that indirect costs (i.e., lost earnings) were only incurred when patients were diagnosed with cancer before the age of 67 years (a conservative estimate) because the median age of retirement in Canada in 2021 was 64.5 years [22]. Regardless of disease status, the average individual over the age of 67 would not be working and would, therefore, not bear any indirect costs during this time. Finally, we applied the average lost earnings per year in the continuing care phase for 2 years as previous literature has shown that 60% of cancer patients return to work within 1 to 2 years after diagnosis [23,24]. Thus, on average, after 2 years in the continuing care phase, cancer patients should not be bearing any indirect costs as they would have returned to work at this point. In total, patients will bear indirect costs for a maximum of 4 years over the life course (i.e., 1 year during the initial care, 2 years during continuing care, and 1 year during terminal care). However, it is important to note that patients would only incur continuing care costs for the duration of time they are alive; for example, if a patient dies from cancer 1.5 years after diagnosis, they will only incur indirect costs during (part of) the initial and terminal care phases.

### 2.5. Base Case Analysis

Table 1 represents the average costs used in the OncoSim model across cancer sites for the base case scenario. The base case scenario represents the model’s expected case or the results that most closely reflect the true value of the economic burden of cancer. Depending on the type of cost, either cancer site-specific estimates or the average costs of multiple cancer sites were inputted into the OncoSim-All Cancers model by phase of care. If the data source only had one cancer site, this estimate was used for all other sites as well. For health system costs, cancer-site and sex-specific costs were used. For indirect costs, cancer sites were divided by high, medium, and low survival-each survival had its respective costs. There were no sex-specific estimates for indirect costs. For OOPCs and time costs, averages were used across cancer sites and sex. With this information, the OncoSim-All Cancers model projected cancer costs in 2021 by phase of care, sex, and cancer site.

### 2.6. Sensitivity Analyses

We estimated the best-case and worst-case scenarios by altering the assumptions used in our base-case analysis. The best-case scenario and worst-case scenario represent the best and worst possible outcomes, respectively. First, we adjusted the number of years patients received medical care during the continuing phase of care since in our base-case analysis our assumption was based on the source study’s observation period (10 years). Second, we varied the cancer diagnosis age cut-off when estimating indirect costs to adjust for differences in retirement age. Third, we varied the hourly wage when estimating time costs as the value of a patient’s time can be conceptualized in several ways. Most studies valued each patient’s time equally by using the median hourly wage however, some analyses have used the “human capital” approach to value a patient’s time based on sex and age-specific wages [12,13,25]. In the sensitivity analyses, we continued to value each patient’s time equally however, the hourly wage was either increased or decreased to represent those with higher and lower-paying jobs. Lastly, OOPCs were adjusted based on a study in Ontario which sampled patients with malignant neoplasms in the terminal treatment phase [26]. The estimates reported here were 40% lower than those used in our base-case analysis. Based on this estimate, we adjusted the initial and terminal phase of care costs since the two treatment phases are similar in duration.

In the worst-case scenario, during the continuing care phase, we assumed patients received additional medical care due to cancer for the rest of their life; in the base case, we assumed that patients received no more than 10 years of additional medical care. For indirect costs, we applied the average of lost earnings per year in the continuing care phase for a maximum of 4 years (versus 2 years in the base case). Here, we also assumed individuals retired at age 70 to represent those who work past the average retirement age. We also increased indirect costs during the initial and terminal care phases by 20% to account for the lost earnings of caregivers. A Canadian study on indirect costs of cancer reported that the annual earnings of spouses of cancer patients decreased by CAD 1500–2000 [18]. Increasing the base case indirect costs by 20% is equivalent to an additional CAD 1200 in costs, which is a conservative estimate. However, this is also assuming that caregivers are spouses only; in reality, caregivers can include a multitude of individuals. For time costs in the worst-case scenario, we used the average Canadian hourly wage in 2021 (CAD 31.14); this estimate reflects patients with higher-paid work [17]. Lastly, OOPCs were increased by 40% for the initial and terminal phase of care.

In the best-case scenario, when estimating indirect costs, we assumed individuals retired at age 60. We estimated time costs using the federal minimum wage (CAD 15.00 an hour); this wage reflects patients who are not in the workforce or have lower-paying jobs [17]. The OOPCs for the initial and terminal phase of care were also decreased by 40%. Cost components that were not discussed in the sensitivity analyses section remained identical to the base-case scenario. See Table 2 for a breakdown of changes between the base-case analysis and the sensitivity analyses. See Supplementary Table S3 for the cost values used in the sensitivity analysis.

## 3. Results

**Figure 4:**
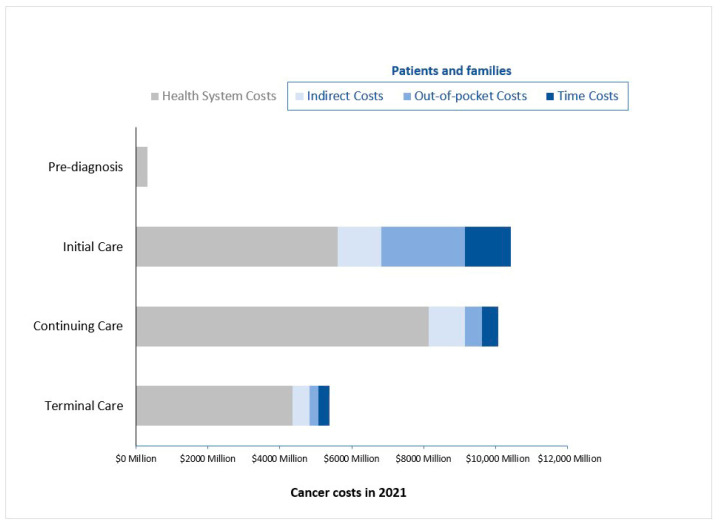
The economic burden of cancer by phase of care in 2021 Costs are rounded and presented in 2021 CAD.

In 2021, the OncoSim-All Cancers model projected around 2,000,000 people ever diagnosed with cancer in Canada. Among them, 225,000 people were newly diagnosed with cancer (i.e., in the initial phase of care), 86,000 were in their terminal phase of care, and the remaining 1,689,000 (85%) were people in the continuing phase of care. From a societal perspective, the projected economic burden of cancer in Canada was CAD 26 billion in 2021; about one-third of the economic burden was borne by patients and families (Figure 4). The economic burden of cancer incurred by patients and families varied across the cancer trajectory, where the largest burden occurred during the first year after diagnosis (i.e., initial phase of care). During this time, patients and families’ cancer-related costs amounted to CAD 4.8 billion, similar to the health systems’ costs of the initial care phase (CAD 5.6 billion). The continuing phase of care was the next phase with the largest burden for patients and families (CAD 1.9 billion). Patients and families experienced the least amount of the economic burden in the terminal phase of care (CAD 1.0 billion). The relative distribution of costs across care categories was similar across three scenarios (base case and sensitivity analyses). See Supplementary Figures S1 and S2.

Across all treatment phases, the total economic burden borne by patients and families was CAD 7.8 billion; out-of-pocket costs totaled CAD 3.1 billion, indirect costs amounted to CAD 2.7 billion and time costs totaled CAD 2.0 billion. However, when sensitivity analyses were performed, the relative distribution of costs borne by patients and families differed (Table 3). In the best-case scenario, out-of-pocket costs were the largest expenses borne by patients and families (CAD 2.1 billion), followed by time costs (CAD 1.6 billion) and indirect costs (CAD 9.4 million). In the worst-case scenario, indirect costs were the largest expense of the burden borne by patients and families (CAD 5.8 billion), followed by out-of-pocket costs (CAD 4.1 billion) and then time costs (CAD 3.7 billion). In this scenario, indirect costs were adjusted to include patients’ and caregivers’ lost earnings. The hourly wage used to calculate time costs was also increased to CAD 31.14 (i.e., a CAD 12 increase). The total societal cost burden decreased by 12% in the best-case scenario while it increased by approximately 39% in the worst-case scenario. Across care categories, indirect and time costs contributed the most to the change observed in the total societal cost burden.

### Cancer Costs by Sex and Cancer Site

From a societal perspective, the top four cancers (lung, breast, colorectal, and prostate) had the highest burden. For indirect costs, breast, leukemia, lung, and brain cancer patients experienced the largest burden. See Table 4 for a distribution of costs by cancer site. The economic burden was similar for both sexes. See Supplementary Figure S3 for the distribution of costs by sex.

## 4. Discussion

This study provides a comprehensive estimate of the economic burden of cancer from a societal perspective. Previous Canadian studies estimated the economic burden of cancer using health system costs only; however, other cost components (i.e., OOPCs, time costs, and indirect costs) are needed to obtain a more comprehensive picture of the total economic burden of cancer [27,28,29,30,31]. Our analysis found that health system costs amounted to CAD 18.4 billion alone; however, after incorporating cancer-related expenses from the patients’ and families’ perspective, the annual economic burden of cancer in Canada increased by CAD 7.8 billion (almost 50% increase). Across all phases of care, OOPCs were the highest component of patients’ and families’ costs. Indirect costs were the second-largest cost incurred by patients and families. The combined effect of lost income and increased expenditure can pose a significant financial barrier in obtaining cancer care, especially for those in lower socioeconomic status [32]. New policy options addressing the affordability of cancer treatments should be considered to prevent additional consequences (i.e., financial strain) of severe illness.

Costs incurred by patients and families were highest in the initial care phase and lowest in the terminal care phase. Patients and families experiencing the smallest burden in the terminal care phase is likely since in the last 12 months of life cancer patients spend more time in the hospital, receiving end of life care. During this time, patients will primarily be using direct health system resources rather than spending their own money on homecare, outpatient drugs, at-home devices, and so on. Overall, our analysis found that the total economic burden in the continuing phase of care was almost as large as those in the initial phase of care-this is mostly attributable to health system costs. This result is likely since 85% of living people ever diagnosed with cancer were in the continuing care phase. Additionally, the duration of the continuing care phase may span several years whereas, the initial and terminal phases of care are only 2 years of the cancer trajectory, collectively. This suggests that long-term complications from initial cancer treatments, prolonged cancer treatments (such as maintenance treatments), follow-up care, and surveillance could have a large impact on the overall economic burden of cancer.

The Economic Burden of Illness in Canada 2005–2008 (EBIC) report found that the economic burden of malignant neoplasms (cancer) amounted to CAD 5.5 billion (2021 CAD). This estimate includes direct healthcare system costs (i.e., medical expenditures) and indirect costs (i.e., lost productivity) for cancer in Canada [27]. Unfortunately, the EBIC report is an underestimate of the true economic burden of cancer as costs of cancer-related care such as chemotherapy and radiation therapy were not included. In our analysis, health system costs alone amounted to CAD 18.4 billion; this suggests that the inclusion of chemotherapy and radiation therapy has a large impact on the economic burden of cancer. Comparing the relative costs of cancer to other conditions and diseases, the EBIC report found that neuropsychiatric conditions are the costliest (CAD 15.6 billion, 2021 CAD) while oral conditions are the least costly (CAD 50.3 million, 2021 CAD). Genitourinary diseases (CAD 4.9 billion), respiratory diseases (CAD 4.6 billion) and parasitic diseases (CAD 3.6 billion) were reported to have a similar range of costs as cancer (costs in 2021 CAD) [27].

To our knowledge, The Annual Report to the Nation on the Status of Cancer in the United States is the first study to estimate the economic burden of cancer from a societal perspective; this report analyzed OOPCs and time costs [13]. This report found that OOPCs were 3–4× as large as time costs while our analysis estimated that OOPCs were 1.5x as large as time costs. This discrepancy in relative cost is likely since the United States does not have universal healthcare; many US citizens are required to pay out-of-pocket for cancer treatment. This report also quantified the patient economic burden of cancer by using a phase-costing approach; the authors found OOPCs, and time costs were highest in patients initially diagnosed [13]. Similarly, our analysis found OOPCs, time costs, as well as indirect costs, were highest during the initial phase of care.

The Canadian Agency for Drugs and Technologies in Health (CADTH) indicates that when conducting economic evaluations from a societal perspective, all relevant costs to the patients, caregivers, and employers must be considered. Our study suggests that patients’ and families’ cost are considerable and varies by phase of care and cancer site. Therefore, to adequately measure the economic burden of cancer from a societal perspective all cost components should be included. These results will help fill the knowledge gaps in respect to the patient economic burden of cancer in Canada.

### 4.1. Strengths and Limitations

This study has a few strengths worth noting; this is the first Canadian study to pool together high-quality data to assess and quantify the economic burden of cancer from a societal perspective (i.e., health system, out-of-pocket, time, and indirect costs). Secondly, this study uses a phase-based costing approach to describe the differences in cancer-related expenses across treatment stages (i.e., initial, continuing, and terminal care). This is important as the cost of cancer varies throughout the course of care hence, more accurate estimates can be obtained by systematically separating each treatment stage. Lastly, this study provides cost estimates for over 30 cancer sites and both sexes.

This study has several limitations. The analysis uses an economic modeling approach to project cancer costs; thus, the validity of the projections depends on the quality of data and the assumptions made when combining data from multiple sources. Although the study was more comprehensive than previous studies, it may still have underestimated the economic burden of cancer for various reasons. First, it included lost earnings of patients only but did include those of their family members/caregivers. Second, it did not include the impact of cancer on patients’ and caregivers’ mental health and quality of life (i.e., psychosocial costs). Third, the model only included cancers diagnosed in adults (that is, patients 18 years and older). Fourth, the net time costs were based on patients older than 65 years of age; younger individuals tend to receive more aggressive care than older patients [33,34]. Fifth, there was no available data for the time and indirect costs of the pre-diagnosis phase to include in the model however, the health system cost estimates suggest that pre-diagnosis phase costs are likely small, relative to other phases of care. Lastly, it did not include costs borne by private third-party health care insurers and by charitable organizations, amounts which in Canada are not trivial. On the other hand, there may been some overlap (and hence double counting) of direct time costs and indirect costs, because some of the time spent receiving, travelling to, and waiting for care also represented time that would otherwise have been spent working and earning income. Additionally, the source study for indirect costs sampled patients aged 25–65 years old, which may have overestimated indirect costs as younger patients require more intensive care.

### 4.2. Future Research

This study provides a high-level estimate of the economic burden of cancer in Canada, capturing the costs from patients and families that are not often included in other studies. Future research could explore the economic burden of cancer for different sub-population, such as cancers that are often diagnosed at a younger age vs. those diagnosed at an older age, or cancers that are more fatal vs. not. To address health inequity, future research should also highlight the economic burden of cancers that are often more common in people with lower socioeconomic status.

## 5. Conclusions

This study provides a comprehensive estimate of the cancer economic burden from a societal perspective, which includes health system costs, out-of-pocket costs, time costs, and indirect costs. The economic burden of cancer in Canada from a societal perspective in 2021 amounted to CAD 26.2 billion. Our findings show that a large proportion of the burden is borne by patients and families (30%), which is often not reported. Studies that only include cancer costs from the health system’s perspective would have missed a considerable portion of cancer costs. These estimates can be used to inform policies concerning health insurance coverage and resource allocation decisions.

## Figures and Tables

**Table 1:** Model inputs-cancer costs per patient by cost type and cancer treatment phase.

	Cancer Treatment Care Phase				
	Pre-Diagnosis (3 Months)	Initial Care (12 Months)	Continuing Care (Annual/12 Months)	Terminal Care (12 Months)	Total
**Direct Health System Costs**	1612 ^a^	28,891 ^a^	6070 ^a^	52,861 ^a^	89,434
**Direct Out-of-Pocket Costs**	0	10,649 ^b^	264 ^c^	2868 ^d^	13,781
**Direct Time Costs**	0	5774 ^e^	375 ^e^	3504 ^e^	9653
**Indirect Costs**	0	5923 ^f^	664 ^f^	5923 ^f^	12,510
**Total**	1612	51,237	7373	65,156	125,378

Cost estimates are presented as mean patient costs in 2021 CAD. Net costs were presented for direct health system costs, direct time costs, and indirect costs. Gross costs were presented for OOPCs. The pre-diagnosis phase is defined as the 3 months before cancer diagnosis and includes costs related to diagnostic testing and hospital admissions. The initial phase of care is defined as 12 months after diagnosis (including date of diagnosis) and includes the costs of primary course of therapy and any adjuvant therapy. The continuing care phase (expressed as an annual cost) encompasses costs with ongoing surveillance and active follow-up treatment for cancer recurrence and/or new primary cancers. The terminal phase of care is defined as the last 12 months before death (which may or may not be due to cancer) captures costs with the intensive services, often palliative in nature. Note, in the OncoSim model, not all individuals who die of cancer were in palliative care. ^a^ = These estimates are obtained from a study that includes phase-specific net costs for over 20 cancer sites. The mean net cost for all cancer sites is presented in the table for simplicity. This value includes direct costs incurred by the public third-party payer (i.e., health system) such as chemotherapy, radiation therapy, physician services, inpatient hospitalizations, etc. ^b^ = This estimate considers breast, colorectal, lung, and prostate cancer patients who have undergone a minimum of 4 weeks of cancer treatment. This estimate was used for the initial care phase and includes costs associated with drugs, homecare, supplies, travel, etc. ^c^ = This estimate considers long-term prostate cancer survivors. This value was used for the continuing care phase and includes costs of medication, equipment, homecare, etc. ^d^ = This estimate considers multiple cancer sites at the terminal stage of treatment (i.e., 12 months prior to death or the end of their participation in the study). This value includes personal expenses related to palliative care. ^e^ = This estimate is derived from a study from the US, which includes colorectal patients and estimates net time per treatment phase. The median Canadian hourly wage was used to determine net time costs. This value includes the cost of round-trip travel to care, waiting, and receiving care. ^f^ = This estimate considers multiple cancer sites for patients aged 25 to 61 years who survived at least 3 years after diagnosis. This estimate was used for the initial, continuing, and terminal care phases. It includes the net lost earnings from employment for patients.

**Table 2:** Cost changes in best-case and worst-case scenarios.

		Pre-Diagnosis	Initial Care	Continuing Care	Terminal Care
Best-case	**Direct Health System Costs**	Base case estimates	Base case estimates	Base case estimates	Base case estimates
	**Direct Out-of-Pocket Costs**	Base case estimates	Estimates were decreased by 40%.	Base case estimates	Estimates were decreased by 40%.
	**Direct Time Costs**	Base case estimates	Hourly wage decreased to CAD 15.00	Hourly wage decreased to CAD 15.00	Hourly wage decreased to CAD 15.00
	**Indirect Costs**	Base case estimates	Base case estimates	Costs were extended for a maximum of two years. For cancer sites where average life expectancies were greater than 60 years, costs were set to zero for individuals over 60.	Base case estimates
Worst-case	**Direct Health System Costs**	Base case estimates	Base case estimates	No time limit on receiving additional medical care (base-case limit is 10 years).	Base case estimates
	**Direct Out-of-Pocket Costs**	Base case estimates	Estimates were increased by 40%.	Base case estimates	Estimates were increased by 40%.
	**Direct Time Costs**	Base case estimates	Hourly wage increased to CAD 31.14.	Hourly wage increased to CAD 31.14.	Hourly wage increased to CAD 31.14.
	**Indirect Costs**	Base case estimates	Estimates were increased by 20%.	Costs were extended for a maximum of four years. For cancer sites where average life expectancies were greater than 70 years, costs were set to zero for individuals over 70.	Estimates were increased by 20%.

**Table 3:** The economic burden of cancer in 2021, base case vs. sensitivity analyses (in 2021 CAD; M represents millions).

		Pre-Diagnosis	Initial Care	Continuing Care	Terminal Care	Total
Worst-Case	Direct Health System Costs	321 M	5599 M	12,575 M	4359 M	22,854 M
	Direct Out-of-Pocket Costs	0	3273 M	491 M	354 M	4118 M
	Direct Time Costs	0	2062 M	1133 M	502 M	3698 M
	Indirect Costs	0	2097 M	2809 M	850 M	5755 M
	Total	321 M	12,096 M	17,008 M	5964 M	35,389 M
Base Case	Direct Health System Costs	321 M	5599 M	8137 M	4359 M	18,415 M
	Direct Out-of-Pocket Costs	0	2338 M	491 M	253 M	3082 M
	Direct Time Costs	0	1267 M	439 M	309 M	2016 M
	Indirect Costs	0	1216 M	1002 M	465 M	2684 M
	Total	321 M	10,420 M	10,069 M	5386 M	26,196 M
Best-Case	Direct Health System Costs	321 M	5599 M	8137 M	4359 M	18,415 M
	Direct Out-of-Pocket Costs	0	1403 M	491 M	152 M	2045 M
	Direct Time Costs	0	993 M	344 M	242 M	1579 M
	Indirect Costs	0	442 M	320 M	174 M	937 M
	Total	321 M	9372 M	9292 M	5028 M	24,013 M

Costs are rounded to the closest million.

**Table 4:** Economic burden of cancer-by-cancer site in 2021 (in 2021 CAD; M represents millions).

	Direct Health System Costs	Direct Out-of-Pocket Costs	Direct Time Costs	Indirect Costs
Oral cavity	211 M	33 M	22 M	40 M
Oropharynx	96 M	16 M	10 M	20 M
Hypopharynx	22 M	3 M	2 M	1 M
Other oral	124 M	18 M	12 M	23 M
Esophagus	293 M	32 M	22 M	28 M
Stomach	377 M	50 M	36 M	47 M
Colorectal	2468 M	364 M	251 M	107 M
Liver	278 M	40 M	27 M	116 M
Pancreas	651 M	78 M	53 M	53 M
Larynx	116 M	17 M	12 M	5 M
Lung	2713 M	419 M	291 M	243 M
Melanoma	363 M	116 M	70 M	121 M
Breast	2141 M	393 M	235 M	417 M
Cervix	81 M	24 M	13 M	29 M
Body of uterus	353 M	99 M	61 M	105 M
Ovary	264 M	40 M	29 M	166 M
Prostate	1514 M	356 M	226 M	99 M
Testis	49 M	19 M	9 M	21 M
Bladder	720 M	161 M	108 M	38 M
Kidney	500 M	98 M	64 M	115 M
Brain/CNS	498 M	42 M	30 M	190 M
Thyroid	248 M	95 M	49 M	96 M
Hodgkin lymphoma	101 M	19 M	9 M	20 M
Non-Hodgkin lymphoma	968 M	134 M	90 M	108 M
Multiple myeloma	510 M	44 M	31 M	38 M
Leukemias	879 M	91 M	62 M	330 M
Vulva	64 M	10 M	7 M	3 M
Vagina	19 M	3 M	2 M	1 M
Anal	68 M	10 M	7 M	13 M
Penile	24 M	3 M	2 M	1 M
Other cancer	1701 M	256 M	175 M	75 M

Costs are rounded to the closest million.

## Data Availability

Detailed data about model input parameters and projections are available on the OncoSim web application via https://www.partnershipagainstcancer.ca/tools/oncosim/ (accessed on 17 March 2022).

## Supplementary Materials

The following supporting information can be downloaded at: https://www.mdpi.com/article/10.3390/curroncol29040223/s1, Table S1: Study descriptions; Table S2: Cost conversions; Table S3: Sensitivity analysis values-Worst case scenario (Best case scenario); Figure S1: Economic burden of cancer in 2021, sensitivity analysis versus base case scenario; Figure S2: Sensitivity analysis results by phase of care; Figure S3: Economic burden of cancer by sex, in 2021.
