# Supplementary material for: The Economic Burden of Cancer in Canada from a Societal Perspective
Source: Curr Oncol. 2022 Apr 14;29(4):2735–48. doi: 10.3390/curroncol29040223 (PMC9025082; doi:10.3390/curroncol29040223)
Supplement: Supplementary file 1 [file curroncol-29-00223-s001.zip › curroncol-1634885-supplementary.pdf]

Current Oncology

MDPI

Supplementary Files

Table S1. Study descriptions

Study	Jurisdiction	Sample size	Definition of cost	Definition of patient population	Cancer site(s)	Corresponding OncoSim phase
**Jeon (2017)**	Canada	2597	**Indirect costs:** net lost earnings per year from employment/ annual earnings	Cancer patients aged 25 to 61 years who survived for at least 3 years after diagnosis	Multiple	Initial, Continuing Care, and Terminal
**Longo (2021)**	Canada	901	**Direct out-of-pocket costs:** self-reported costs including drugs, homecare, homemaking, complementary/ alternative medicines, vitamins/supplements, family care, accommodations, devices, travel, parking, and "other" costs	Patients with a minimum of 4 weeks of cancer treatment (ideally still on active treatment)	Breast, Colorectal, Lung, Prostate	Initial
**de Oliveira (2013)**	Ontario	585	**Direct out-of-pocket costs:** self-reported cost spent visiting health professionals, medication, equipment, community services, household help, and time lost from work and leisure	Cancer patient survivors diagnosed in 1993-1994, 1997-1998 and 2001-2002	Prostate	Continuing Care
**Dumont (2015)**	Canada	205	**Direct out-of-pocket costs:** personal expenses related to palliative care	Cancer patients enrolled in a regional palliative care program	Multiple	Terminal
**Yabroff (2005)**	USA	74,413	**Direct time costs:** net costs of time spent traveling to and from care, waiting for appointments, and receiving care	Cancer patients aged 65 years or older at some time between 1995-1998	Colorectal	Initial, Continuing Care, Terminal (phase-specific estimates)
**de Oliveira (2016)**	Ontario	394,092	**Direct health system costs:** net costs from the perspective of the public third-party payer	Adult patients diagnosed with a primary cancer between 1997-2007 and who survived 30 days or more after diagnosis	Multiple	Pre-diagnosis, Initial, Continuing Care, Terminal (phase-specific estimates)

Table S2. Cost conversions

Study	Cost type (treatment phase)	Original estimate	CPI Scalar	Adjusted value (2021 CAD, per year)	Notes
**Jeon (2017)**	Indirect (initial, continuing, terminal)	Initial/Terminal<br>High Survival: $4,048<br>Middle Survival: $4,376<br>Low Survival: $11,660<br><br>Continuing<br>High Survival: $2,947<br>Middle Survival: $3,055<br>Low Survival: $11,209<br><br>(2010 CAD, per year)	CPI 2021<br>143.9<br><br>CPI 2010<br>117.4<br><br>Scalar<br>1.23	Initial/Terminal<br>High Survival: $4,962<br>Middle Survival: $5,363<br>Low Survival: $14,291<br><br>Continuing<br>High Survival: $3,612<br>Middle Survival: $3,745<br>Low Survival: $13,739	Original estimates were reported as "time since diagnosis (T+ x year)." T+1 was used as the initial and terminal phase. An average of T+2 and T+3 was used for the continuing phase.
**Dumont (2015)**	Out-of-pocket (terminal)	$1,812 per 6-months (rural)<br><br>$1,267 per 6-months (urban)<br><br>(2010 CAD)	CPI 2021<br>143.9<br><br>CPI 2010<br>117.4<br><br>Scalar<br>1.23	$3,359	Weighted average based on the Canadian population was taken (rural = 19%, urban = 81%). Costs were then converted to a yearly estimate by multiplying the bi-yearly estimate by 2.
**de Oliveira (2013)**	Out-of-pocket (continuing)	$200 per year (2006 CAD)	CPI 2021<br>143.9<br><br>CPI 2006<br>109.0<br><br>Scalar<br>1.32	$264	-

**Longo (2021)**	Out-of-pocket (initial)	$781 per 28 days (2020 CAD)	CPI 2021<br>143.9<br><br>CPI 2020<br>137.5<br><br>Scalar<br>1.05	$10,649	Average cost per day was calculated then converted into a yearly estimate by multiplying the daily estimate by 365.
**Yabroff (2005)**	Time (initial, continuing, terminal)	Initial<br>301.61 hours per phase<br><br>Continuing<br>1.63 hours per month<br><br>Terminal<br>183.01 hours per phase	-	Initial<br>$5,774<br><br>Continuing<br>$375<br><br>Terminal<br>$3,504	Hours were taken from a USA study and multiplied by median hourly wage in Canada 2021 ($19.14). Continuing care costs were converted to an annual estimate by multiplying the monthly estimate by 12.
**de Oliveira (2016)**	Health system (pre-diagnosis, initial, continuing, terminal)	Reported 21 cancer site-specific estimates.	CPI 2021<br>143.9<br><br>CPI 2016<br>129.1<br><br>Scalar<br>1.12	Average pre-diagnosis<br>$1,611<br><br>Average initial<br>$28,891<br><br>Average continuing<br>$6,070<br><br>Average terminal<br>$52,861	Cancer site specific estimates were used for each phase.

Table S3. Sensitivity analysis values – Worst case scenario (Best case scenario).

	Cancer Treatment Care Phase			
	Pre-diagnosis (3 months)	Initial care (12 months)	Continuing care (annual/12 months)	Terminal care (12 months)
**Indirect Costs**	$0.00 ($0.00)	$7,107.68 ($5,923.07)	$1,328.16 ($664.08)	$7,107.68 ($5,923.07)
**Direct Out-of-Pocket Costs**	$0.00 ($0.00)	$14,908.50 ($6,389.36)	$264.04 ($264.04)	$4,014.57 ($1,720.53)
**Direct Time Costs**	$0.00 ($0.00)	$9,393.14 ($4,524.14)	$609.16 ($293.40)	$5,699.54 ($2,745.15)
**Direct Health System Costs**	$1,611.51 ($1,611.51)	$28,890.69 ($28,890.69)	$7,665.10 ($6,070.30)	$52,861.32 ($52,861.32)

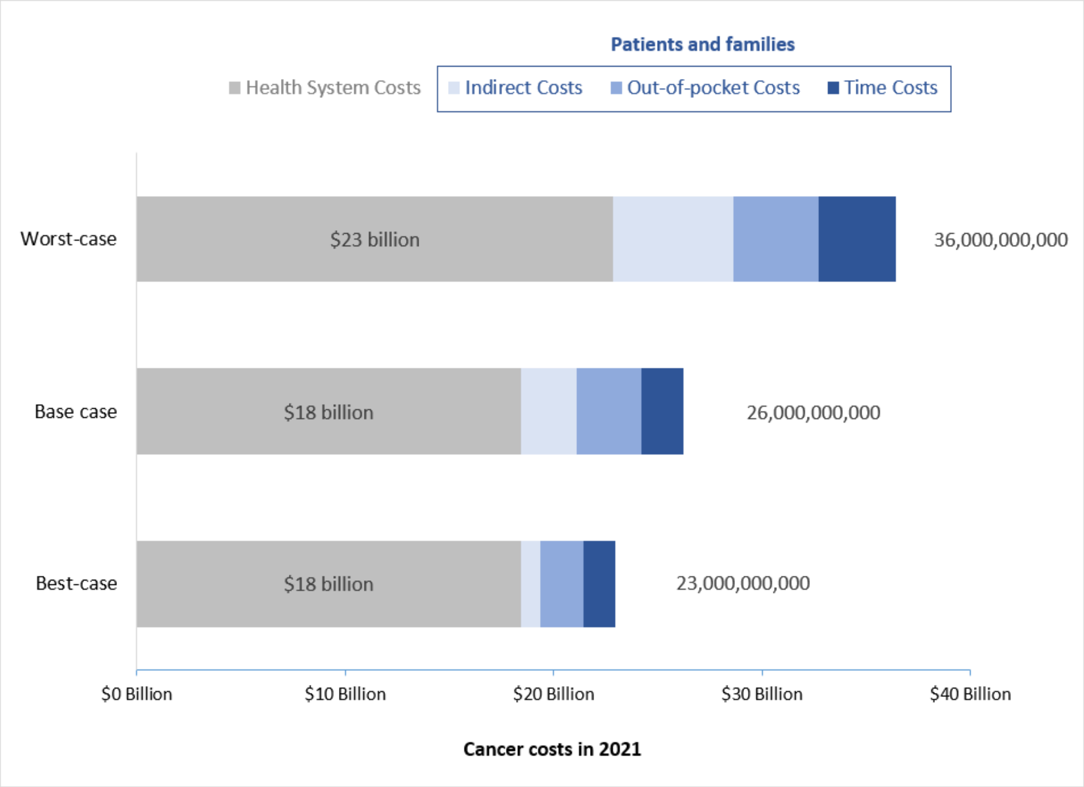

Figure S1. Economic burden of cancer in 2021, sensitivity analyses versus base case scenario. Costs are rounded and presented in 2021 $CAD.

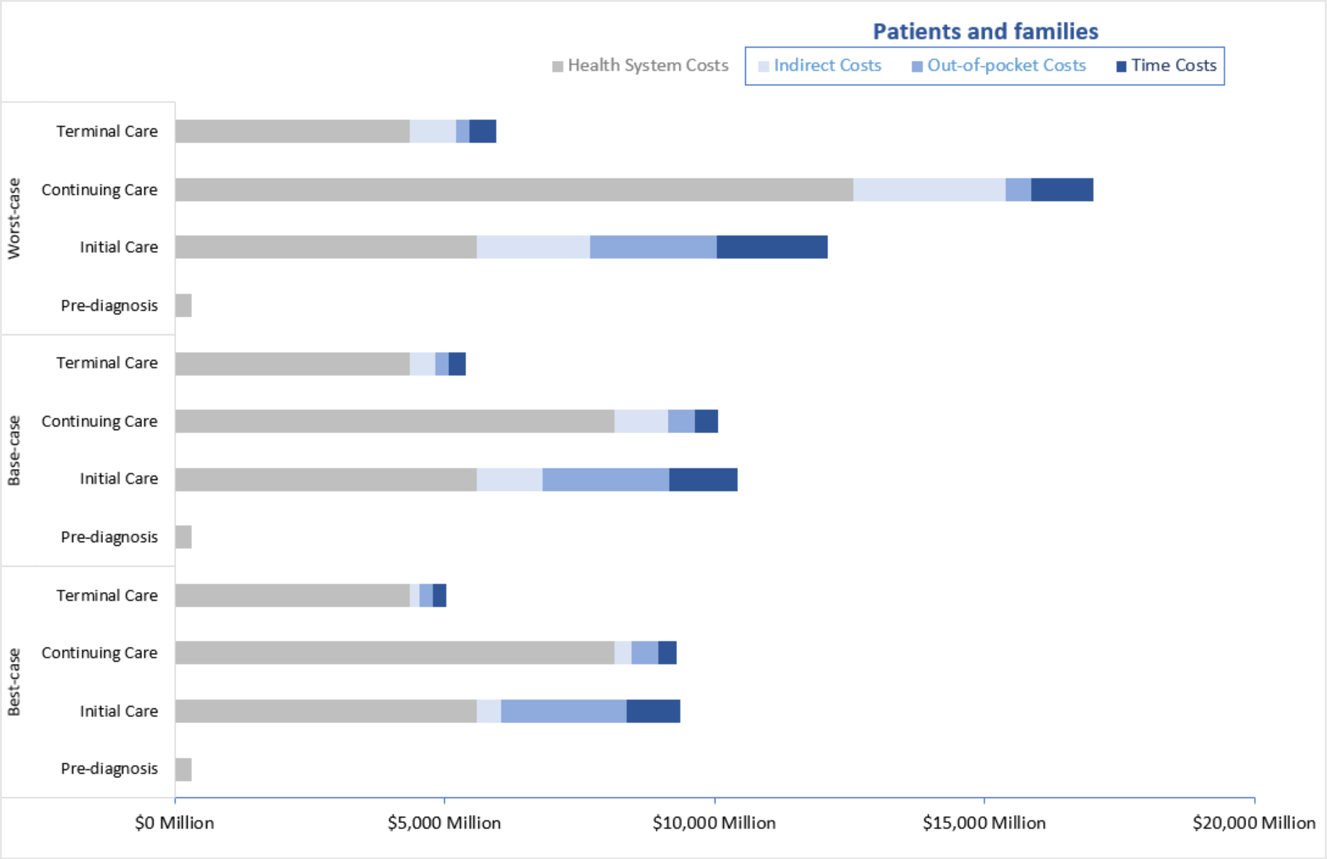

Figure S2. Sensitivity analysis results by phase of care. Costs are rounded and presented in 2021 $CAD.

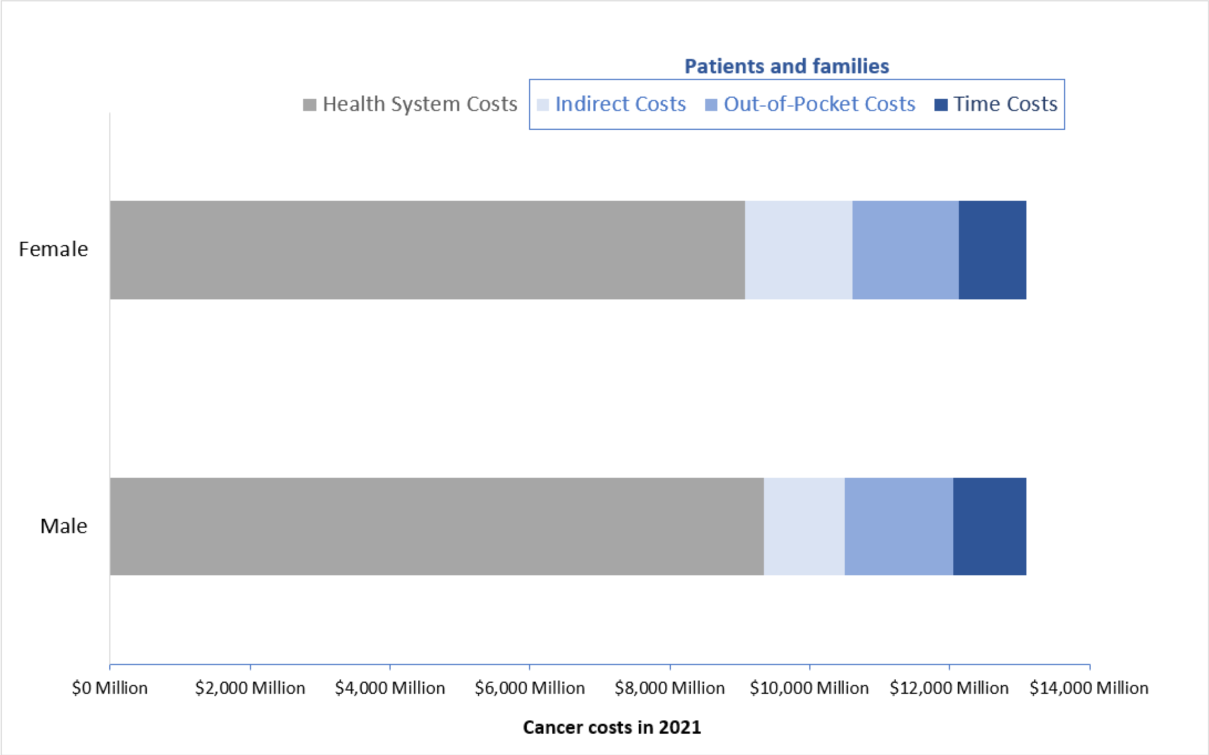

Figure S3. Economic burden of cancer by sex, in 2021. Costs are rounded and presented in 2021 $CAD.
